# Role of the Iodide–Methylammonium Interaction in the Ferroelectricity of CH_3_NH_3_PbI_3_

**DOI:** 10.1002/anie.201910599

**Published:** 2019-11-12

**Authors:** J. Breternitz, F. Lehmann, S. A. Barnett, H. Nowell, S. Schorr

**Affiliations:** ^1^ Department Structure and Dynamics of Energy Materials Helmholtz-Zentrum Berlin für Materialien und Energie Hahn-Meitner-Platz 1 14109 Berlin Germany; ^2^ Institute of Chemistry Universität Potsdam 14469 Potsdam Germany; ^3^ Diamond Light Source Didcot OX11 0DE UK; ^4^ Department of Geosciences Freie Universität Berlin Malteserstrasse 74–100 122449 Berlin Germany

**Keywords:** ferroelectricity, hybrid perovskites, inorganic chemistry, photovoltaic materials, structure elucidation

## Abstract

Excellent conversion efficiencies of over 20 % and facile cell production have placed hybrid perovskites at the forefront of novel solar cell materials, with CH_3_NH_3_PbI_3_ being an archetypal compound. The question why CH_3_NH_3_PbI_3_ has such extraordinary characteristics, particularly a very efficient power conversion from absorbed light to electrical power, is hotly debated, with ferroelectricity being a promising candidate. This does, however, require the crystal structure to be non‐centrosymmetric and we herein present crystallographic evidence as to how the symmetry breaking occurs on a crystallographic and, therefore, long‐range level. Although the molecular cation CH_3_NH_3_^+^ is intrinsically polar, it is heavily disordered and this cannot be the sole reason for the ferroelectricity. We show that it, nonetheless, plays an important role, as it distorts the neighboring iodide positions from their centrosymmetric positions.

## Graphical abstract

**Cause and effect**: Although the question of the ferroelectricity of CH_3_NH_3_PbI_3_ is of essential importance for understanding its performance, it has long been disputed. Insight into the crystallographic origin of the breaking of the inversion symmetry in the structure, a necessary prerequisite for ferroelectricity, is now provided.

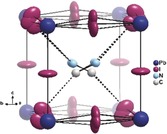

## Introduction

It is clear that hybrid perovskites have changed the way we look at solar absorber materials.^1^, ^2^, ^3^, ^4^ Traditionally, semiconductors were thought to be rigid solids with highly defined atom positions. Hybrid perovskites, however, were shown to have a high defect tolerance^5^ and a flexible crystal structure, with remarkable positional freedom of the molecular cation^6^, ^7^ and ionic movement.^8^, ^9^ This latter feature makes the reliable determination of a crystal structure challenging, as the average long‐range order no longer reflects all the properties of the material. It is probably also for this reason that no real consensus has been reached as to whether CH_3_NH_3_PbI_3_ is centrosymmetric or not at room temperature.^10^ Although many bulk and thin‐film measurements indicate that CH_3_NH_3_PbI_3_ shows a ferroelectric effect under ambient conditions,^11^, ^12^, ^13^, ^14^ other studies either could not reproduce this effect or came to a different conclusion.^15^, ^16^, ^17^, ^18^ Besides the direct observation of a ferroelectric response, there is a crystallographic prerequisite for ferroelectricity: the crystal structure must be polar, that is, not only belong to a space group that is non‐centrosymmetric, but must also belong to one of the 10 polar crystal classes.^19^ The commonly accepted crystal structure of CH_3_NH_3_PbI_3_ at room temperature, however, belongs to the space group *I*4/*mcm*,^10^, ^20^ which is centrosymmetric and hence would not allow any of the above‐mentioned effects. Herein, we set out to conduct high‐resolution single‐crystal diffraction to elaborate the reason for the observed polarizability of CH_3_NH_3_PbI_3_ combined with a discussion regarding the possible space group of the compound.

## Results and Discussion

**Figure 1:**
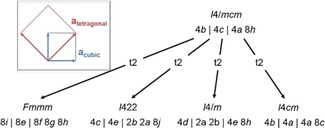
Group/subgroup relationships between the common space group *I*4/*mcm* and further space groups found in the literature. Inset: relationship of the lattice vectors in the *ab*‐plane between the cubic aristotype and the tetragonal setting.

Although *I*4/*mcm* is the commonly chosen space group for CH_3_NH_3_PbI_3_, numerous other choices are documented in the literature (Figure 1). These choices roughly fall into two categories: 1) space groups that no longer contain the *c*‐glide plane^21^, ^22^, ^23^ and 2) the space group *I*4*cm*,^24^ which is the only polar maximal subgroup of *I*4/*mcm*. It is possible to refine the crystal structure in any of these space groups: since all the alternative choices are subgroups of the common choice *I*4/*mcm*, they all contain a subset of symmetry elements, but no symmetry elements that would not exist in *I*4/*mcm*. Therefore, a crystal structure in *I*4/*mcm* must also contain all the symmetry elements of the lower symmetry hettotypes. When comparing the atomic parameters between the different refinements, however, it becomes evident that all the refined structures are closely related to each other.

In fact, the space groups falling in the first category were chosen by the original authors because they observed supplementary reflections, which violate the systematic extinctions dictated by the translational symmetry element, the *c*‐glide plane.^21^ However, this apparent symmetry breaking is most probably a result of twinning of the single crystals in these studies and is aggravated through the nature of the material: the cubic‐to‐tetragonal transition in the system occurs relatively close to room temperature, thus signifying that the energy difference between the two phases under ambient conditions is marginal. Therefore, one could easily assume that the crystal nucleation points form in the cubic symmetry and only the bulk material is tetragonal. If this was the case, the choice of the *c*‐axis out of the three equivalent axes in the cubic system is arbitrary and could easily change within a crystal and one would expect axis twins. Although CH_3_NH_3_PbI_3_ at room temperature is tetragonal, the mismatch between the crystallographic *c‐axis* on the one hand and the crystallographic *a‐* and *b*‐axes (with *a*=*b*) on the other hand is less than 1 %. This is not directly visible when looking at the lattice constants, because the tetragonal lattice constants are related to the cubic ones through *a*_tetragonal_=√2⋅*a*_cubic_ (including a 45° shift, see Figure 1) and *c*_tetragonal_=2⋅*c*_cubic_. Axis twinning would, therefore, not necessarily result in extensive peak splitting or supplementary reflections, as the twinned reflections almost perfectly overlap with the main reflections, apart from the positions where the systematic extinctions of the main reflections should lie. Including the appropriate twin law in the refinement of the data provided in the original data^21^, ^22^ suppresses the systematic extinction violations entirely and hence supports the explanation of apparent extinction violation through twinning effects (see the Supporting Information for detailed analysis). Furthermore, the splitting of the Pb or I positions induced by the choice of this subgroup is not reflected in the atomic positions. In fact, since the space groups under discussion possess a different translational symmetry to *I*4/*mcm*, they should also show supplementary reflections in the powder diffraction spectrum,^25^ but no such supplementary reflections were documented.

The situation for the second category (see above) is different: *I*4*cm* does not add additional splitting to the atomic positions, but allows more positional freedom for the atomic positions. The most striking difference between *I*4/*mcm* and *I*4*cm* is the lack of a mirror plane perpendicular to the fourfold axis, that is, in the *ab*‐plane. This allows the atomic positions to move arbitrarily along the crystallographic *c*‐axis and, hence, allows a shift of the atoms outside a common plane. Such a shift induces a permanent polar moment and, hence, can induce ferroelectricity. Although the molecular cation CH_3_NH_3_^+^ is intrinsically polar, it is dynamically and statically disordered^26^ and, therefore, probably does not induce an effective macroscopic moment. To explain this evident mismatch with the experimental evidence for ferroelectricity, we performed high‐resolution synchrotron single‐crystal diffraction to study the atomic positions with the best accuracy possible.

Conventionally, ferroelectric perovskites show a shift of the cations.^27^ This shift can be very small indeed, as was recently shown in the ferroelectric phase of SrTiO_3_.^28^ Therefore, we performed single‐crystal diffraction at the Pb L‐absorption edges. Under these conditions, the complex part of the atomic structure factor is maximal and can become non‐negligible. Therefore, Friedel's law is not strictly valid any more. Briefly: as the intensity of the reflections is proportional to the square of the atomic structure factors, it will be equal for *hkl* and *h̄k̄l̄* reflections. When the complex part of the structure factor becomes non‐negligible, this is no longer true and the observation of such Bijvoet pairs would be a direct proof of a non‐centrosymmetric structure. We did not, however, directly observe the breaking of Friedel's law, but this is most likely an effect of inversion twinning in the crystals under consideration, rather than a clear indication of centrosymmetry. In fact, several recent studies have observed twin domains in CH_3_NH_3_PbI_3_ crystals, both in thin films and bulk crystals.^29^, ^30^, ^31^

**Figure 2:**
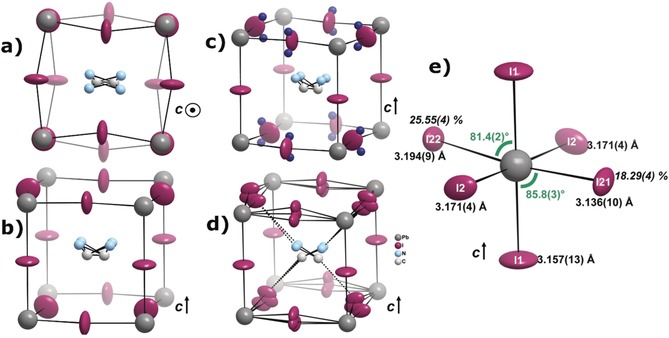
Structural peculiarities of CH_3_NH_3_PbI_3_ at room temperature. a) Orientation of the CH_3_NH_3_^+^ cation in the pseudo‐cubic [PbI_3_] cage along the *c*‐axis and b) in a general section in a conventional 2 I‐site refinement. c) Illustration of the highest residual electron density peaks in the 2 I‐site model (dark blue dots) and d) the cage, including the split‐iodine positions. e) Representation of the PbI_6_ octahedron including the split sites. Pb−I distances are given in black, the relative occupancies of I21 and I22 in italics, and the I1‐Pb‐I21/I22 angles in green.

Using the space group *I*4*cm* instead of *I*4/*mcm* during the crystal‐structure refinement yielded a refinement that is very similar to the structures reported in the literature, without a clear shift of the relevant atoms (see the Supporting Information). However, two features are distinctly different to the refinements in *I*4/*mcm*: 1) the orientation of the molecular cation is less disordered than in previous studies^10^, ^20^, ^32^ and only shows two distinct molecule directions with four orientations, as two N positions lie close to each other (Figure 2 a,b). 2) The highest residual electron density peaks (+3.24 e^−^ Å^−3^) in the system are close to I2, which is the iodine site within the *ab*‐plane. The differences between the orientation of the molecular cation in this refinement and previous studies is only seemingly contradictive: the supplementary positions found in the previous studies aiming to elucidate the molecular orientation are a direct consequence of the higher symmetry in *I*4/*mcm*. These studies were based on powder diffraction, but the differences between *I*4/*mcm* and *I*4*cm* are invisible in powder diffraction, as the different reflections perfectly overlap with each over. We note that the C and N attributions are arbitrary, as they can hardly be distinguished by X‐ray diffraction because of their similar electron count and the time and space averaging of the diffraction method. Therefore, more elaborate analyses of the orientation of the molecular cation are better performed using other techniques.^7^, ^20^, ^33^, ^34^ As a consequence of Fourier truncation and incomplete absorption correction, one normally observes residual electron density peaks, that is, deviations from the model observed electron density, close to the heaviest atoms (here Pb), as their intensity is roughly linked to the electron count.^35^, ^36^ Having them at the iodine position instead is probably due to the missing assignment of some electron density. Indeed, this can be easily interpreted as partial occupation of iodine distributed over three atomic sites, of which two are outside the *ab*‐plane. It should be noted that similar residual electron density peaks can be found in the datasets of Jaffe et al.^21^ and Arakcheeva et al.^22^

The effects, discussed in (1) and (2) above, are in fact related to each other and probably influence each other. It is clear that the molecular cation is roughly pointing towards two of the iodine atoms at opposite edges of the roughly cube‐shaped cage (Figure 2). This is easily understandable as this orientation maximizes the hydrogen‐bonding interaction between the molecular cation and the surrounding iodine atoms.

On the other hand, the iodine atoms positioned out of the plane are shifted in such a way that they specifically approach the molecular cations at the edges the cations point towards, while they are shifted away at the other edges (Figure 2 d). This is in line with the general argument of maximizing X−H⋅⋅⋅I (X=C, N) interactions, as it allows shorter H−I distances. Furthermore, the shifting is observed above and below the *ab*‐plane, but the vector between the two shifted positions is not perpendicular to the *ab*‐plane but stands at an angle of 62.5° to the plane. This is tremendously important, as a perpendicular distortion would still be explainable in *I*4/*mcm*, while such a shifted situation clearly is not and, hence, explains the non‐centrosymmetric arrangement of CH_3_NH_3_PbI_3_ at room temperature. It should be noted that this is an indicator of the crystallographic symmetry breaking but does not necessarily dictate the polar axis. In fact, Leonhard et al. pointed out recently that the material is polarized in the direction of the crystallographic *c*‐axis,^37^ in line with the polar axis of space group *I*4*cm*.

Clearly, the arrangement of the inorganic framework and the arrangement of the molecular cation influence each other in this compound and one might ask the question as to whether the molecular arrangement causes the iodine shift or whether the iodine shift causes a locking of the molecular cation. The consequences of the iodine shifting on the arrangement in the [PbI_6_] octahedra (Figure 2 e) is not great but distinctive: the Pb−I distances become more anisotropic but the differences are generally below 0.1 Å and the I‐Pb‐I angles are slightly distorted from the ideal 90° arrangement. Using diffraction techniques, it is impossible to answer this question directly because of the time and space averaging in diffraction. However, the great strength of this explanation is that it does not solely rely on the intrinsic polarity of the molecular cation, which is both statically and dynamically disordered, but also its relationship with the surrounding iodine atoms. In fact, no matter how the individual molecular cation is ordered in each individual cage, its relationship with the iodine atoms will be similar so that the shift of the iodine atoms remain relatively constant, thereby creating macroscopic polarity in the compound. Although the results presented herein were obtained from single crystals to profit from the higher resolution of X‐ray diffraction, the same relationship between the molecular cation and inorganic backbone is also probable in thin films, with ferroelectricity effectively being observed therein.^37^

## Conclusions

This finding is of crucial importance for understanding hybrid perovskites: it not only makes the finding of ferroelectric effects in CH_3_NH_3_PbI_3_ at ambient conditions reasonable, but it effectively explains where they come from: the interaction of the molecular cation with the anion framework. Therefore, the unique properties of hybrid perovskites critically depend on the nature of the organic cation. It will be an important next step to extend this study to variable temperatures and cation mixtures to elucidate their effect on the relationship between fundamental properties and performance. This study also points to why all‐inorganic perovskites do not exhibit the same efficiencies as hybrid perovskites. This finding raises the fundamental question as to whether the desired effects of the molecular cation, in particular the high efficiency, can be preserved while targeting its negative features, especially the operation stability under light, or whether this poses a critical intrinsic dilemma of these compounds that cannot be overcome.

## Experimental Section

Crystals were grown at room temperature according to the antisolvent vapour method described by Rakita et al.^12^ Single‐crystal X‐ray diffraction was conducted on beamline I19 (EH1) at the Diamond Light Source synchrotron using the programs Jana2006,^38^ APEX3,^39^ cbf_to_sfrm.py,^40^ and SHELXL2017‐1^41^ for data treatment.

Refinement values for the final split‐site model: Space group: *I*4*cm*; *Z=*4; *ρ*=4.171 g mL^−1^; *V=*987.16(8) Å^3^; *a=*8.8438(3) Å; *c=*12.6215(5) Å; *μ*=26.39 mm^−1^; −12 ≤ *h* ≤12; −12 ≤ *k* ≤12; −17 ≤ *l* ≤17; *R*_int_ *=*0.054; *R*_1_ (*I*>2*σ*)=0.035; *wR*_2_ (all)=0.113; *GoF*=1.19; −1.06 ≤ Δe^−^ Å^−13^ ≤2.03. Further refinement details may be found in the Supporting Information.

## Conflict of interest

The authors declare no conflict of interest.

## Supporting information

As a service to our authors and readers, this journal provides supporting information supplied by the authors. Such materials are peer reviewed and may be re‐organized for online delivery, but are not copy‐edited or typeset. Technical support issues arising from supporting information (other than missing files) should be addressed to the authors.

Supplementary
