# Supplementary material for: Role of the Iodide–Methylammonium Interaction in the Ferroelectricity of CH3NH3PbI3
Source: Angew Chem Int Ed Engl. 2019 Nov 12;59(1):424–8. doi: 10.1002/anie.201910599 (PMC6972664; doi:10.1002/anie.201910599)
Supplement: Supplementary file 1 — Supplementary [file ANIE-59-424-s001.pdf]

Angewandte Chemie
Eine Zeitschrift der Gesellschaft Deutscher Chemiker

Supporting Information

Role of the Iodide–Methylammonium Interaction in the Ferroelectricity of $CH_3NH_3PbI_3$

J. Breternitz, F. Lehmann, S. A. Barnett, H. Nowell, and S. Schorr*

anie_201910599_sm_miscellaneous_information.pdf

1. Twinning in Arakcheeva et al. (21) and Jaffe et al. (20)

To test for possible twinning as cause for the supposedly observed breaking of the c-glide plane, a twin-law according to pseudo-merohedral pseudo-cubic axial twinning using the command:

```
TWIN 0.5 0.5 -0.5 0.5 0.5 0.5 1 -1 0
```

According to a 90° rotation around the <110> direction in the tetragonal unit cell.

SHELXL list file output for the untwinned model in Arakcheeva et al. (21) using *I4cm*:

```
   h    k    l         Fo^2      Sigma    Why rejected (first 50 of each listed)
   0    5    7         0.08      0.01     systematically absent but >3sig(I)

       35  Systematically absent reflections rejected


      773  Reflections read, of which      35  rejected

  -8 =< h =<  9,      0 =< k =< 13,      0 =< l =< 13,   Max. 2-theta =   62.53

        1  Systematic absence violations (I>3sig(I)) before merging

       13  Inconsistent equivalents

      432  Unique reflections, of which       0  suppressed

 R(int) = 0.0083     R(sigma) = 0.0050      Friedel opposites not merged

 Maximum memory for data reduction =      987 /     5340


 Number of data for d > 0.659A (CIF: max) and d > 0.833A (CIF: full)
 (ignoring systematic absences):
 Unique reflections found (point group)        432     254
 Unique reflections possible (point group)    1004     510
 Unique reflections found (Laue group)         432     254
 Unique reflections possible (Laue group)      524     269
 Unique Friedel pairs found                      0       0
 Unique Friedel pairs possible                 480     241
```

SHELXL list file output for the twinned model in Arakcheeva et al. using *I4cm* (Approximate twin fraction 1%):

```
      773  Reflections read, of which       0  rejected

  -8 =< h =<  9,      0 =< k =< 13,      0 =< l =< 13,   Max. 2-theta =   62.53

        0  Systematic absence violations (I>3sig(I)) before merging

       13  Inconsistent equivalents

      462  Unique reflections, of which       0  suppressed

 R(int) = 0.0083     R(sigma) = 0.0056      Friedel opposites not merged

 Maximum memory for data reduction =      988 /     5620
```

```
Number of data for d > 0.659A (CIF: max) and d > 0.833A (CIF: full)
(ignoring systematic absences):
Unique reflections found (point group)        748     413
Unique reflections possible (point group)    1004     510
Unique reflections found (Laue group)         443     256
Unique reflections possible (Laue group)      524     269
Unique Friedel pairs found                    305     157
Unique Friedel pairs possible                 480     241
```

The raw hkl data from Jaffe *et al.* was brought in the right setting for *I4cm* using Jana2006 (32).

SHELXL list file output for the untwinned model in Jaffe et al. (20) using *I4cm*:

```
  h   k   l        Fo^2      Sigma    Why rejected (first 50 of each listed)
  0   3  -1        6.96      0.50     systematically absent but >3sig(I)
  0   3   1        7.72      0.44     systematically absent but >3sig(I)
  0   3  -1        8.62      0.50     systematically absent but >3sig(I)
  0   3   1        7.66      0.38     systematically absent but >3sig(I)
  0   3   1        7.85      0.40     systematically absent but >3sig(I)
  0   3  -1        7.00      0.45     systematically absent but >3sig(I)
  0   3   1        7.43      0.42     systematically absent but >3sig(I)
  0   3   1        7.91      0.52     systematically absent but >3sig(I)
  0   3  -1        8.21      0.44     systematically absent but >3sig(I)
  0   3  -1        7.51      0.36     systematically absent but >3sig(I)
  0   3  -1        7.72      0.39     systematically absent but >3sig(I)
  0   3   1        8.22      0.48     systematically absent but >3sig(I)
  0   3   1        7.21      0.38     systematically absent but >3sig(I)
  0   5  -1        1.24      0.25     systematically absent but >3sig(I)
  0   5   1        0.62      0.17     systematically absent but >3sig(I)
  0   5   1        0.71      0.20     systematically absent but >3sig(I)
  0   5  -1        1.12      0.28     systematically absent but >3sig(I)
  0   5   1        0.96      0.18     systematically absent but >3sig(I)
  0   5   1        0.60      0.13     systematically absent but >3sig(I)
  0   5  -1        0.52      0.16     systematically absent but >3sig(I)
  0   5   1        0.88      0.22     systematically absent but >3sig(I)
  0   5   1        0.90      0.19     systematically absent but >3sig(I)
  0   5  -1        0.98      0.15     systematically absent but >3sig(I)
  0   5   1        0.80      0.24     systematically absent but >3sig(I)
  0   5  -1        0.95      0.17     systematically absent but >3sig(I)
  0   5  -1        0.84      0.24     systematically absent but >3sig(I)
  0   7   1        0.57      0.18     systematically absent but >3sig(I)
  0   7   1        0.83      0.23     systematically absent but >3sig(I)
  0   1  -3        7.87      0.41     systematically absent but >3sig(I)
  0   1   3        8.67      0.44     systematically absent but >3sig(I)
  0   1  -3        7.42      0.52     systematically absent but >3sig(I)
  0   1  -3        8.43      0.39     systematically absent but >3sig(I)
  0   1   3        9.29      0.44     systematically absent but >3sig(I)
  0   1   3        8.76      0.40     systematically absent but >3sig(I)
  0   1   3        8.12      0.56     systematically absent but >3sig(I)
  0   5  -3        6.82      0.59     systematically absent but >3sig(I)
  0   5  -3        5.59      0.50     systematically absent but >3sig(I)
  0   5   3        6.75      0.49     systematically absent but >3sig(I)
  0   5  -3        4.72      0.49     systematically absent but >3sig(I)
  0   5   3        6.84      0.41     systematically absent but >3sig(I)
  0   5  -3        7.62      0.53     systematically absent but >3sig(I)
  0   5  -3        6.79      0.49     systematically absent but >3sig(I)
  0   5   3        5.70      0.47     systematically absent but >3sig(I)
  0   1   5        0.44      0.14     systematically absent but >3sig(I)
  0   1  -5        0.82      0.22     systematically absent but >3sig(I)
  0   1   5        0.42      0.09     systematically absent but >3sig(I)
```

```
  0   1   5        0.44      0.13     systematically absent but >3sig(I)
  0   1   5        0.31      0.10     systematically absent but >3sig(I)
  0   3  -5       10.01      0.71     systematically absent but >3sig(I)
  0   3  -5       10.92      0.57     systematically absent but >3sig(I)

** etc. **

     393  Systematically absent reflections rejected


    7624  Reflections read, of which     393  rejected

-13 =< h =< 14,    -14 =< k =< 14,    -20 =< l =< 20,   Max. 2-theta =   70.06

      76  Systematic absence violations (I>3sig(I)) before merging

      21  Inconsistent equivalents

    1280  Unique reflections, of which       0  suppressed

R(int) = 0.0628     R(sigma) = 0.0375      Friedel opposites not merged

Maximum memory for data reduction =      975 /    16887


Number of data for d > 0.600A (CIF: max) and d > 0.833A (CIF: full)
(ignoring systematic absences):
Unique reflections found (point group)       1280     494
Unique reflections possible (point group)    1291     496
Unique reflections found (Laue group)         669     262
Unique reflections possible (Laue group)      671     262
Unique Friedel pairs found                    611     232
Unique Friedel pairs possible                 620     234
```

SHELXL list file output for the twinned model in Jaffe et al. using *I4cm* (Approximate twin fraction 12%):

```
    7624  Reflections read, of which       0  rejected

-13 =< h =< 14,    -14 =< k =< 14,    -20 =< l =< 20,   Max. 2-theta =   70.06

       0  Systematic absence violations (I>3sig(I)) before merging

      21  Inconsistent equivalents

    1385  Unique reflections, of which       0  suppressed

R(int) = 0.0633     R(sigma) = 0.0385      Friedel opposites not merged

Maximum memory for data reduction =      988 /    17823


Number of data for d > 0.596A (CIF: max) and d > 0.833A (CIF: full)
(ignoring systematic absences):
Unique reflections found (point group)       1284     494
Unique reflections possible (point group)    1316     496
Unique reflections found (Laue group)         673     262
Unique reflections possible (Laue group)      684     262
Unique Friedel pairs found                    611     232
Unique Friedel pairs possible                 632     234
```

2. Experimental details

Crystals were grown at room temperature according to the antisolvent vapor method described by Rakita et al. PbI_2 (99 %, ACROS Organics), ethyl acetate (ChemCruz, HPLC grade), acetonitrile (Sigma-Aldrich, 99.5 %), diethyl ether (Merck, 99.7 %), methylammonium iodide (Sigma-Aldrich, 98 %) and HI solution (stabilized 57 wt.-%in H_2O, 99.95 %, Sigma-Aldrich) were used as supplied. Both, crystals grown with diethyl ether and ethyl acetate as antisolvent were tested, but those grown using ethyl acetate generally exhibited better crystal quality and the study was conducted on a crystal of this series. It should be noted that we did not find a single crystal which did not show any signs of twinning and finally selected one that appeared least twinned for the subsequent detailed analysis. Crystals were prepared in an Ar-filled glovebox and covered in oil during the measurement to avoid sample decomposition due to moisture. It should be emphasized that crystals with approx. edge lengths of 20 µm were used for these experiments to avoid further complications with heavy twinning and strong absorption.

Single-crystal X-ray diffraction was conducted at the I19 beamline at the Diamond Light Source synchrotron. Using the double crystal monochromator of the beamline, the X-ray energy was adjusted between 12.97 keV and 15.3 keV, i.e. in proximity of the L-III and L-II absorption edges of lead. This was chosen as the initial approach of this experiment was to test, whether a breaking of Friedel's law as direct proof for the lack of inversion symmetry could be observed. It should be emphasized that we did not observe any significant breaking of Friedel's law, which is most probably due to the domain nature of the crystals. In fact, we refined the final model as inversion twin yielding in a twin fraction of 48 %. In order to observe Friedel pairs, one would probably need to align the domains, for instance through crystallization in an electric field. We are currently testing such possibilities. Given no direct observation of Friedel pairs could be achieved, further analysis was performed on the highest measured energy: 15.3 keV (λ = 0.81036 Å).

Reflections were measured using a Pilatus 2M detector. Data integration and Lorentz factor correction (using SAINT V8.38A) and absorption correction (using SADABS-2016/2) were performed using the Bruker APEX3 suite (33), for which the Pilatus CBF format was converted to SFRM using a custom built program by Natalie Johnson and Mike Probert (34). The authors are thankful for their kind help with this. The latter was done using a semi-empirical multiscan absorption correction as the crystal form could not be reliably determined given the size and the covering in oil. Refinements were performed using SHELXL2013 (35).

The C-N distance of the $CH_3NH_3^+$ cation was fixed to 1.47 Å as common for the molecular cation (19). The split iodine sites in the split-site model were constrained to have equal displacement parameters. This refinement was further damped at the later stages since the molecular cation is heavily disordered. It should be noted that the assignment of carbon and nitrogen in the model is arbitrary, since the small difference in electron density between carbon and nitrogen makes them literally indistinguishable, particularly in connection with iodine and lead.

3. Refinement without split site model

Table S1: Crystal data

CH_6I_3NPb	*Z* = 4

M_r = 619.96	F(000) = 1040
Tetragonal, *I*4*cm*	D_x = 4.171 Mg m^{-3}
a = 8.8438 (3) Å	μ = 26.39 mm^{-1}
c = 12.6215 (5) Å	T = 293 K
V = 987.16 (8) Å^3	

Table S2: Data collection

5668 measured reflections	θ_{max} = 34.5°, θ_{min} = 3.7°
768 independent reflections	h = -12→12
674 reflections with $I > 2\sigma(I)$	k = -12→12
R_{int} = 0.054	l = -17→17

Table S3: Refinement

Refinement on F^2	H-atom parameters not defined
Least-squares matrix: full	$w = 1/[\sigma^2(F_o^2) + (0.0569P)^2 + 22.4079P]$ where $P = (F_o^2 + 2F_c^2)/3$
$R[F^2 > 2\sigma(F^2)]$ = 0.044	$(\Delta/\sigma)_{max}$ < 0.001
$wR(F^2)$ = 0.126	$\Delta\rangle_{max}$ = 3.24 e Å^{-3}
S = 1.15	$\Delta\rangle_{min}$ = -1.34 e Å^{-3}
768 reflections	Absolute structure: Flack x determined using 291 quotients [(I+)-(I-)]/[(I+)+(I-)] (Parsons, Flack and Wagner, Acta Cryst. B69 (2013) 249-259).
20 parameters	Absolute structure parameter: 0.48 (3)
2 restraints	

Table S4: Fractional atomic coordinates and isotropic or equivalent isotropic displacement parameters (Å^2)

	x	y	z	U_{iso}*/U_{eq}	Occ. (<1)
Pb	0.000000	0.000000	0.00012 (2)	0.0298 (3)	
I1	0.000000	0.000000	0.2500 (4)	0.0812 (10)	
I2	0.2135 (2)	0.7135 (2)	0.0007 (7)	0.0844 (8)	
N	0.404 (7)	0.036 (8)	0.278 (6)	0.05 (2)*	0.25
C	0.537 (4)	-0.037 (4)	0.229 (5)	0.048 (15)*	0.5

Table S5: Atomic displacement parameters (Å^2)

	U^{11}	U^{22}	U^{33}	U^{12}	U^{13}	U^{23}
Pb	0.0311 (4)	0.0311 (4)	0.0273 (5)	0.000	0.000	0.000

I1	0.1123 (16)	0.1123 (16)	0.0190 (9)	0.000	0.000	0.000
I2	0.0705 (9)	0.0705 (9)	0.1121 (17)	0.0480 (10)	0.010 (3)	0.010 (3)

Table S6: Geometric parameters (Å, º)

Pb—I1	3.154 (5)	N—C^{vi}	0.81 (7)
Pb—$I1^{i}$	3.157 (5)	N—N^{vii}	0.74 (14)
Pb—$I2^{ii}$	3.1600 (4)	N—C	1.47 (3)
Pb—$I2^{iii}$	3.1600 (4)	N—N^{viii}	1.65 (12)
Pb—$I2^{iv}$	3.1600 (4)	C—C^{vi}	0.92 (10)
Pb—$I2^{v}$	3.1600 (4)		
I1—Pb—$I1^{i}$	180.0	C^{vi}—N—N^{vii}	63 (5)
I1—Pb—$I2^{ii}$	89.87 (16)	C^{vi}—N—C	34 (6)
$I1^{i}$—Pb—$I2^{ii}$	90.13 (16)	N^{vii}—N—C	75 (3)
I1—Pb—$I2^{iii}$	89.87 (16)	C^{vi}—N—N^{viii}	63 (7)
$I1^{i}$—Pb—$I2^{iii}$	90.13 (16)	N^{vii}—N—N^{viii}	90.002 (11)
$I2^{ii}$—Pb—$I2^{iii}$	179.7 (3)	C—N—N^{viii}	29 (3)
I1—Pb—$I2^{iv}$	89.87 (16)	C^{vi}—C—N^{vi}	117 (7)
$I1^{i}$—Pb—$I2^{iv}$	90.13 (16)	C^{vi}—C—N^{viii}	117 (7)
$I2^{ii}$—Pb—$I2^{iv}$	90.000 (1)	N^{vi}—C—N^{viii}	55 (10)
$I2^{iii}$—Pb—$I2^{iv}$	90.000 (1)	C^{vi}—C—N	29 (3)
I1—Pb—$I2^{v}$	89.87 (16)	N^{vi}—C—N	101 (9)
$I1^{i}$—Pb—$I2^{v}$	90.13 (16)	N^{viii}—C—N	88 (9)
$I2^{ii}$—Pb—$I2^{v}$	90.000 (1)	C^{vi}—C—N^{vii}	29 (3)
$I2^{iii}$—Pb—$I2^{v}$	90.000 (1)	N^{vi}—C—N^{vii}	88 (9)
$I2^{iv}$—Pb—$I2^{v}$	179.7 (3)	N^{viii}—C—N^{vii}	101 (9)
Pb—I1—Pb^{ix}	180.0	N—C—N^{vii}	29 (6)
Pb^{x}—I2—Pb^{xi}	163.36 (10)		

Symmetry codes: (i) -*x*, *y*, *z*-1/2; (ii) *y*-1, -*x*, *z*; (iii) -*y*+1, *x*, *z*; (iv) *x*, *y*-1, *z*; (v) -*x*, -*y*+1, *z*; (vi) -*x*+1, -*y*, *z*; (vii) -*y*+1/2, -*x*+1/2, *z*; (viii) *y*+1/2, *x*-1/2, *z*; (ix) -*x*, *y*, *z*+1/2; (x) -*x*+1/2, *y*+1/2, *z*; (xi) *x*, *y*+1, *z*.

4. Split site model refinement

Refined as a 2-component inversion twin with a twin fraction of

Table S7: Crystal data

CH_6I_3NPb	$Z = 4$
$M_r = 619.96$	$F(000) = 1040$
Tetragonal, *I*4*cm*	$D_x = 4.171$ Mg m^{-3}
$a = 8.8438$ (3) Å	$\mu = 26.39$ mm^{-1}
$c = 12.6215$ (5) Å	$T = 293$ K
$V = 987.16$ (8) Å^3	

Table S8: Data collection

5668 measured reflections	$\theta_{max} = 34.5°$, $\theta_{min} = 3.7°$
768 independent reflections	$h = -12 \rightarrow 12$
674 reflections with $I > 2\sigma(I)$	$k = -12 \rightarrow 12$
$R_{int} = 0.054$	$l = -17 \rightarrow 17$

Table S9: Refinement

Refinement on F^2	H-atom parameters not defined
Least-squares matrix: full	$w = 1/[\sigma^2(F_o^2) + (0.0592P)^2 + 5.3133P]$ where $P = (F_o^2 + 2F_c^2)/3$
$R[F^2 > 2\sigma(F^2)] = 0.035$	$(\Delta/\sigma)_{max} = 0.002$
$wR(F^2) = 0.113$	$\Delta\rangle_{max} = 2.03$ e Å^{-3}
$S = 1.19$	$\Delta\rangle_{min} = -1.06$ e Å^{-3}
768 reflections	Absolute structure: Refined as an inversion twin.
27 parameters	Absolute structure parameter: 0.49 (3)
2 restraints	

Table S10: Fractional atomic coordinates and isotropic or equivalent isotropic displacement parameters (Å^2)

	x	*y*	*z*	U_{iso}*/U_{eq}	Occ. (<1)
Pb	0.000000	0.000000	-0.0026 (5)	0.0300 (2)	
I1	0.000000	0.000000	0.2473 (9)	0.0814 (8)	
I2	0.2078 (4)	0.7078 (4)	0.000000	0.0549 (8)	0.5616 (4)
I21	0.2442 (11)	0.7442 (11)	-0.0206 (10)	0.0549 (8)	0.1829 (4)
I22	0.2145 (10)	0.7145 (10)	0.0353 (8)	0.0549 (8)	0.2555 (4)
N	0.420 (6)	0.046 (6)	0.275 (4)	0.044 (13)*	0.25
C	0.540 (3)	-0.040 (3)	0.221 (5)	0.064 (14)*	0.5

Table S11: Atomic displacement parameters (Å^2)

	U^{11}	U^{22}	U^{33}	U^{12}	U^{13}	U^{23}
Pb	0.0314 (3)	0.0314 (3)	0.0273 (4)	0.000	0.000	0.000
I1	0.1128 (13)	0.1128 (13)	0.0187 (7)	0.000	0.000	0.000
I2	0.0570 (7)	0.0570 (7)	0.051 (2)	0.0348 (8)	0.010 (2)	0.010 (2)
I21	0.0570 (7)	0.0570 (7)	0.051 (2)	0.0348 (8)	0.010 (2)	0.010 (2)
I22	0.0570 (7)	0.0570 (7)	0.051 (2)	0.0348 (8)	0.010 (2)	0.010 (2)

Table S12: Geometric parameters (Å, º)

Pb—I21^{i}	3.1359 (11)	Pb—I2^{ii}	3.1711 (9)
Pb—I21^{ii}	3.1359 (11)	Pb—I2^{iii}	3.1711 (9)
Pb—I21^{iii}	3.1359 (11)	Pb—I2^{iv}	3.1711 (9)
Pb—I21^{iv}	3.1359 (11)	Pb—I22^{ii}	3.194 (2)
Pb—I1	3.154 (7)	Pb—I22^{i}	3.194 (2)
Pb—I1^{v}	3.157 (7)	N—N^{vi}	0.43 (12)
Pb—I2^{i}	3.1711 (9)		
I21^{i}—Pb—I21^{iii}	89.70 (4)	I21^{ii}—Pb—I2^{iv}	98.3 (3)
I21^{ii}—Pb—I21^{iii}	89.70 (4)	I21^{iii}—Pb—I2^{iv}	171.0 (3)
I21^{i}—Pb—I21^{iv}	89.70 (4)	I21^{iv}—Pb—I2^{iv}	9.5 (2)
I21^{ii}—Pb—I21^{iv}	89.70 (4)	I1—Pb—I2^{iv}	89.42 (11)
I21^{iii}—Pb—I21^{iv}	171.7 (5)	I1^{v}—Pb—I2^{iv}	90.58 (11)
I21^{i}—Pb—I1	94.2 (3)	I2^{i}—Pb—I2^{iv}	89.994 (2)
I21^{ii}—Pb—I1	94.2 (3)	I2^{ii}—Pb—I2^{iv}	89.994 (2)
I21^{iii}—Pb—I1	94.2 (3)	I2^{iii}—Pb—I2^{iv}	178.8 (2)
I21^{iv}—Pb—I1	94.2 (3)	I1—Pb—I22^{ii}	81.40 (19)
I1—Pb—I1^{v}	180.0	I1^{v}—Pb—I22^{ii}	98.60 (19)
I1—Pb—I2^{i}	89.42 (11)	I2^{i}—Pb—I22^{ii}	170.7 (3)
I1^{v}—Pb—I2^{i}	90.58 (11)	I2^{ii}—Pb—I22^{ii}	8.15 (19)
I1—Pb—I2^{ii}	89.42 (11)	I2^{iii}—Pb—I22^{ii}	88.4 (3)
I1^{v}—Pb—I2^{ii}	90.58 (11)	I2^{iv}—Pb—I22^{ii}	91.4 (3)
I2^{i}—Pb—I2^{ii}	178.8 (2)	I1—Pb—I22^{i}	81.40 (19)
I21^{i}—Pb—I2^{iii}	98.3 (3)	I1^{v}—Pb—I22^{i}	98.60 (19)
I21^{ii}—Pb—I2^{iii}	81.8 (3)	I2^{i}—Pb—I22^{i}	8.15 (19)
I21^{iii}—Pb—I2^{iii}	9.5 (2)	I2^{ii}—Pb—I22^{i}	170.7 (3)
I21^{iv}—Pb—I2^{iii}	171.0 (3)	I2^{iii}—Pb—I22^{i}	91.4 (3)
I1—Pb—I2^{iii}	89.42 (11)	I2^{iv}—Pb—I22^{i}	88.4 (3)
I1^{v}—Pb—I2^{iii}	90.58 (11)	Pb—I1—Pb^{vii}	180.0
I2^{i}—Pb—I2^{iii}	89.994 (2)	Pb^{viii}—I2—Pb^{ix}	160.82 (19)
I2^{ii}—Pb—I2^{iii}	89.994 (2)	Pb^{ix}—I21—Pb^{viii}	171.3 (5)

I21^{i}—Pb—I2^{iv}	81.8 (3)	Pb^{viii}—I22—Pb^{ix}	156.4 (4)

Symmetry codes: (i) *y*-1, -*x*, *z*; (ii) -*y*+1, *x*, *z*; (iii) -*x*, -*y*+1, *z*; (iv) *x*, *y*-1, *z*; (v) -*x*, *y*, *z*-1/2; (vi) -*y*+1/2, -*x*+1/2, *z*; (vii) -*x*, *y*, *z*+1/2; (viii) -*x*+1/2, *y*+1/2, *z*; (ix) *x*, *y*+1, *z*.
